# Comparison of Radiographic Outcomes Between Vertical and Horizontal Screw Configurations in Four-Corner Arthrodesis for Wrists With Scaphoid Nonunion Advanced Collapse or Scapholunate Advanced Collapse

**DOI:** 10.7759/cureus.117748

**Published:** 2026-10-10

**Authors:** Veeranon Chunyawongsak, Mason Tantitham, Nuttapol Khajonvittayakul, Arkaphat Kosiyatrakul, Kittiwan Supichyangur

**Affiliations:** 1 Hand and Reconstructive Microsurgery Unit, Department of Orthopedic Surgery, College of Medicine, Rangsit University, Rajavithi Hospital, Bangkok, THA; 2 Department of Hand and Microsurgery, Tharongchang Hospital, Suratthani, THA; 3 Department of Orthopedics, Phramongkutklao College of Medicine, Phramongkutklao College of Medicine, Bangkok, THA

**Keywords:** four-corner arthrodesis, headless compression screw, radiographic union, scaphoid nonunion advanced collapse, scapholunate advanced collapse

## Abstract

Introduction

Scaphoid nonunion advanced collapse (SNAC) and scapholunate advanced collapse (SLAC) are common patterns of progressive wrist arthritis. Scaphoid excision combined with four-corner arthrodesis (FCA) is a well-established motion-preserving procedure for stage II-III disease. While headless compression screw (HCS) fixation is widely used, the optimal screw configuration remains undefined.

Materials and methods

This retrospective cohort study evaluated patients with SNAC or SLAC wrists who underwent scaphoid excision and FCA with HCS fixation at a tertiary referral center between January 1, 2015, and December 31, 2024. Patients were categorized into two groups according to screw configuration: vertical or horizontal. The primary outcome was time to radiographic union. Secondary outcomes included union rate, carpal height ratio (CHR), joint space height ratio (JSHR), and postoperative complications.

Results

Sixteen patients were included (eight patients in each group). All patients achieved radiographic union (100.0%). The mean time to union was 43.48 ± 12.91 days in the vertical group and 40.63 ± 11.70 days in the horizontal group, demonstrating no statistically significant difference (mean difference: 2.85 days, 95% CI: -10.42 to 16.12; t = -0.46, p = 0.66). Postoperative CHR and JSHR were maintained in both groups, with no significant differences between groups. No cases of hardware failure, infection, neurovascular injury, or reoperation occurred during follow-up.

Conclusions

No statistically significant differences in radiographic outcomes, time to union, or postoperative complication rates were observed between vertical and horizontal HCS configurations in FCA among patients with SNAC and SLAC wrists. These preliminary findings suggest that both orientations are feasible and can be selected based on patient carpal anatomy and surgeon preference.

## Introduction

Scapholunate advanced collapse (SLAC) wrist and scaphoid nonunion advanced collapse (SNAC) wrist represent two of the most well-recognized patterns of progressive post-traumatic degenerative arthritis of the wrist. SLAC wrist results from chronic insufficiency of the scapholunate interosseous ligament, leading to a predictable cascade of degenerative changes [1]. The SNAC wrist, first reported by Fisk in 1970 and later characterized by several investigators, develops from untreated scaphoid nonunion, with progressive carpal collapse and radioscaphoid arthrosis. Despite differences in underlying pathogenesis, both entities show a similar course of progressive joint deterioration and loss of function, including worsening pain, decreased grip strength, and stiffness [2-5].

Because the radiolunate articulation is usually spared even in the later stages of both SLAC and SNAC wrists, motion-preserving salvage procedures remain the treatment of choice over total wrist arthrodesis for most patients [6,7]. Four-corner arthrodesis (FCA) following scaphoid excision, which fuses the capitate, lunate, hamate, and triquetrum, is widely regarded as the standard surgical treatment for patients presenting with stage II-III disease [8,9]. This technique reliably relieves pain while preserving a degree of wrist motion and carpal height, with functional results that generally surpass those of proximal row carpectomy in younger, high-demand patients [10,11].

Various fixation devices have been employed to achieve arthrodesis in FCA, including Kirschner wires (K-wires), staples, dorsal circular plates, and headless compression screws (HCS) [12-15]. Among the available fixation methods, HCS fixation has gained popularity because the implant is completely buried beneath the subchondral bone surface, reducing hardware prominence, preserving articular cartilage, and providing direct interfragmentary compression across the arthrodesis surfaces to achieve stable fixation [16,17]. Across all fixation techniques, the radiographic union rate is approximately 97%, with no significant differences detected across fixation methods. Studies using HCS fixation report union rates as high as 98%, further demonstrating the efficacy of this implant [18].

Despite the widespread use of HCS in FCA, the optimal spatial configuration for screw placement remains undefined. Clinically, two principal screw configurations are used. In the vertical configuration, parallel screws are placed from proximal to distal, usually providing independent fixation of the capitate-to-lunate and triquetrum-to-hamate interfaces [12,15]. In contrast, the horizontal screw configuration uses crossing or divergent screws placed obliquely, with one screw passing from the triquetrum across the hamate into the capitate and the second inserted from the capitate into the lunate [19]. However, no direct comparison of these two HCS configurations has reported radiographic union rates or postoperative complications. Published studies have evaluated each technique independently and reported consistently high radiographic union rates across fixation methods. These findings make it difficult to distinguish between techniques when union rate alone is used as the primary outcome measure. Because the time to radiographic union may influence the duration of postoperative immobilization, rehabilitation timing, and the extent of muscle atrophy and soft tissue contracture, time to union represents a more sensitive and clinically relevant outcome measure than union rate alone.

Therefore, this retrospective cohort study aimed to compare time to radiographic union between vertical and horizontal HCS configurations in patients undergoing FCA for stage II-III SLAC or SNAC wrist. Secondary objectives were to evaluate radiographic union rates, preservation of carpal height and radiolunate joint space as assessed by the carpal height ratio (CHR) and joint space height ratio (JSHR), respectively, and postoperative complications. We hypothesized that the different screw paths and compression vectors achieved with each configuration would produce a measurable difference in time to radiographic union.

## Materials and methods

Study design and ethical approval

The Institutional Review Board of Rajavithi Hospital approved this retrospective comparative study (approval number: 177/2568; approval date: September 30, 2025). Because this was a retrospective review of medical records, informed consent was waived. This study was performed in accordance with the 1964 Declaration of Helsinki and its later amendments [20].

Patient selection and grouping

Medical records and radiographic data were retrospectively reviewed for all patients who underwent scaphoid excision and FCA with HCS fixation for SNAC or SLAC wrist between January 1, 2015, and December 31, 2024, at Rajavithi Hospital. Inclusion criteria were (1) radiologically confirmed stage II-III SNAC or SLAC wrist; (2) age 18 years or older; (3) scaphoid excision combined with FCA using HCS fixation; and (4) complete, assessable postoperative radiographic follow-up. Patients were excluded if they had undergone prior surgery on the operative wrist, had congenital upper-extremity abnormalities, or had pre-existing neurological injury involving the affected extremity. Additional exclusion criteria included systemic disorders that could impair bone healing (such as rheumatoid arthritis, metabolic bone disease, or chronic corticosteroid therapy), as well as incomplete clinical records or insufficient radiographic follow-up.

A total of 16 eligible patients were classified into vertical screw configuration group (n = 8) or the horizontal screw configuration group (n = 8) according to the fixation technique used. Baseline demographics, diagnosis type, disease stage, operative side, and follow-up duration were comparable between the two groups.

Surgical technique and postoperative care

All procedures were performed by experienced hand surgeons under general anesthesia or regional brachial plexus block with the patient placed in the supine position and a pneumatic tourniquet applied to the upper arm. A dorsal longitudinal incision was centered over the radiocarpal and midcarpal joints. The extensor retinaculum was incised, and the extensor tendons were carefully retracted. A dorsal capsulotomy was performed to expose the carpal bones and midcarpal articulation. Partial wrist denervation was routinely performed through excision of the posterior interosseous nerve. The scaphoid was completely excised while preserving the surrounding ligamentous structures. Residual cartilage and fibrous tissue at the capitate-lunate, hamate-triquetrum, and midcarpal interfaces were debrided until healthy cancellous surfaces were achieved. Morselized autologous cancellous bone graft harvested from the excised scaphoid was packed into the prepared fusion sites (Figure 1).

**Figure 1:**
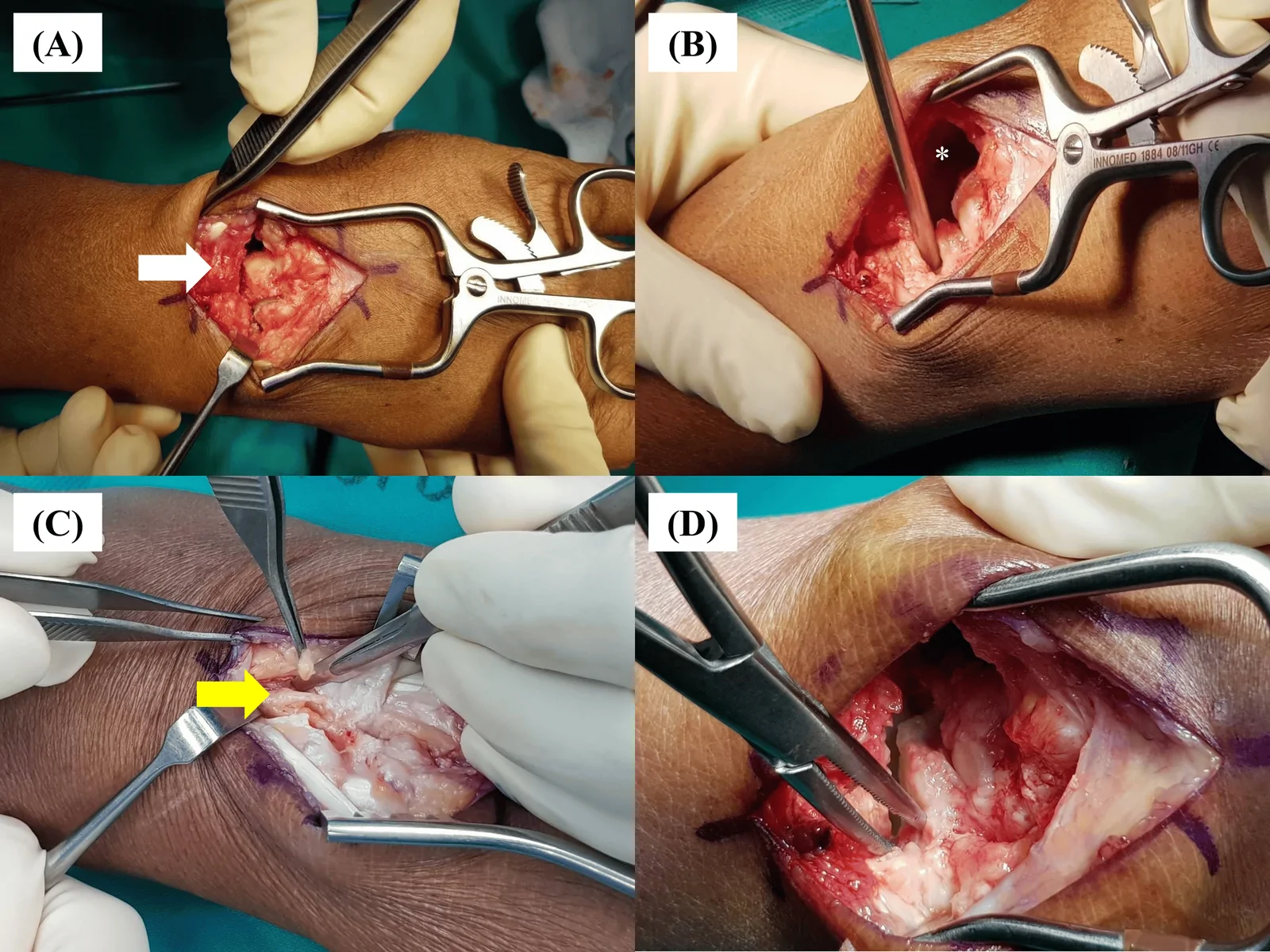
Intraoperative photographs demonstrating the step-by-step surgical exposure and preparation for four-corner arthrodesis (A) Exposure of the midcarpal articulation through a dorsal longitudinal incision; incision of the extensor retinaculum (white arrow) with retraction of the extensor tendons, followed by dorsal capsulotomy. (B) Complete excision of the scaphoid (white asterisk indicates the void after excision) while preserving surrounding ligamentous structures. (C) Partial wrist denervation performed via excision of the terminal posterior interosseous nerve (yellow arrow). (D) Debridement of residual cartilage at the capitate-lunate and hamate-triquetrum interfaces to expose healthy cancellous bone, followed by packing of autologous morselized bone graft harvested from the excised scaphoid. Image Credit: Created by the authors using PowerPoint (Microsoft Corp., Redmond, WA, USA)

Temporary K-wire fixation was applied under fluoroscopic guidance to restore lunate alignment and carpal height before definitive fixation. All fixations utilized 2.4-mm cannulated titanium HCS (Medartis AG, Basel, Switzerland) inserted over dedicated guide wires. In the vertical configuration group, we advanced one HCS antegrade from the lunate into the capitate. A second screw was inserted vertically from the triquetrum into the hamate, maintaining a parallel orientation (Figure 2). In the horizontal configuration group, one HCS was placed from the capitate into the lunate, whereas the second was advanced from the triquetrum across the hamate toward the capitate in a horizontal orientation (Figure 3). All screw heads were countersunk completely below the subchondral articular surface under biplanar fluoroscopy. Anatomical repair of the capsule and extensor retinaculum was completed, and the incision was closed in layers. A short-arm splint was maintained for four to six weeks. Serial outpatient clinical and radiographic examinations were conducted at two, six, 10, 14, and 24 weeks postoperatively until radiographic union was documented. Active wrist range-of-motion exercises were initiated following confirmation of radiographic union.

**Figure 2:**
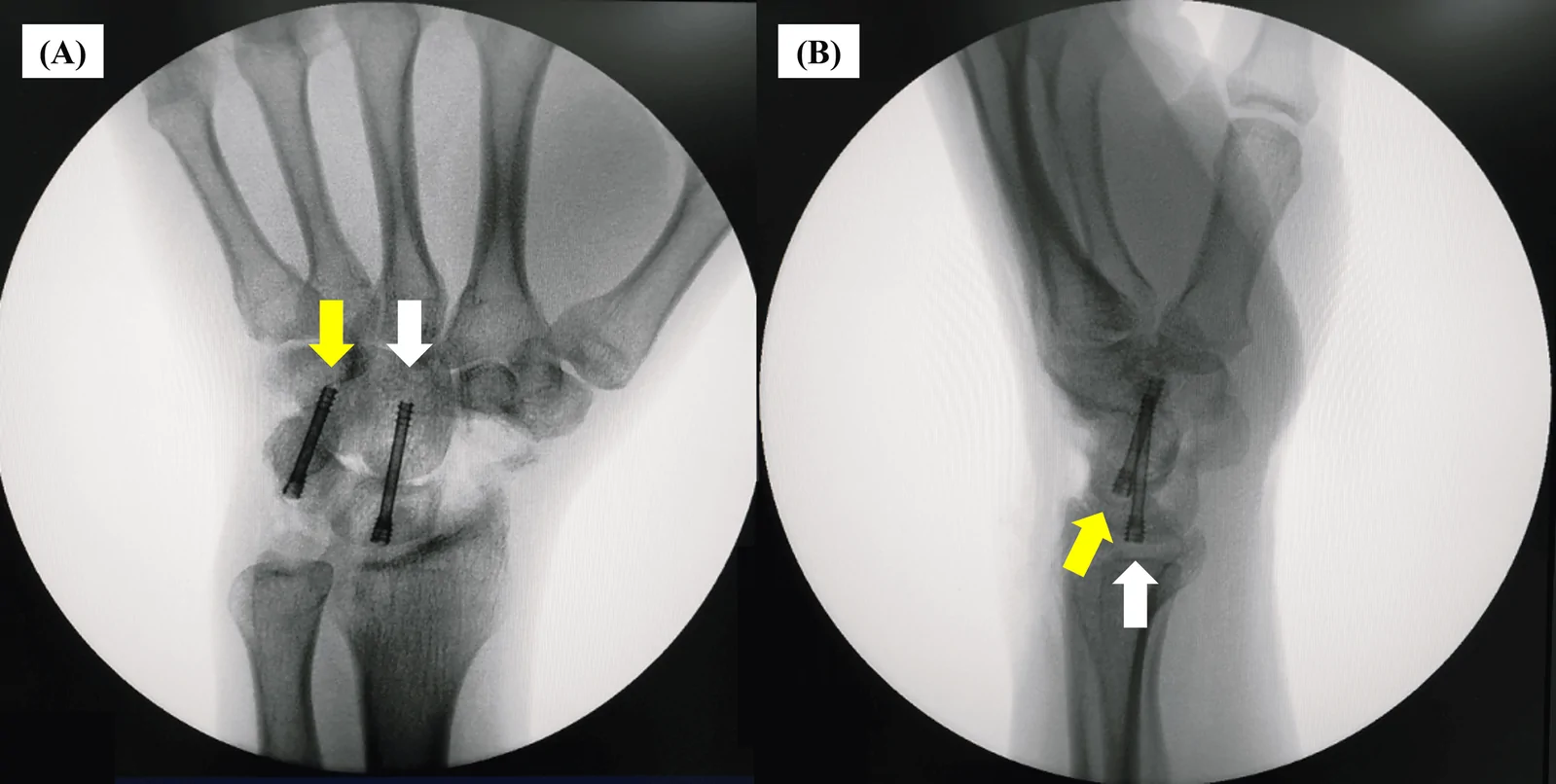
Intraoperative fluoroscopy showing HCS placement in the vertical configuration group Posteroanterior (A) and lateral (B) fluoroscopic views demonstrate anatomical carpal reduction and stable fixation. The white arrow indicates the antegrade HCS advanced from the lunate into the capitate. The yellow arrow indicates the second HCS placed vertically from the triquetrum into the hamate, maintaining a parallel vertical trajectory. HCS: headless compression screw Image Credit: Created by the authors using PowerPoint (Microsoft Corp., Redmond, WA, USA)

**Figure 3:**
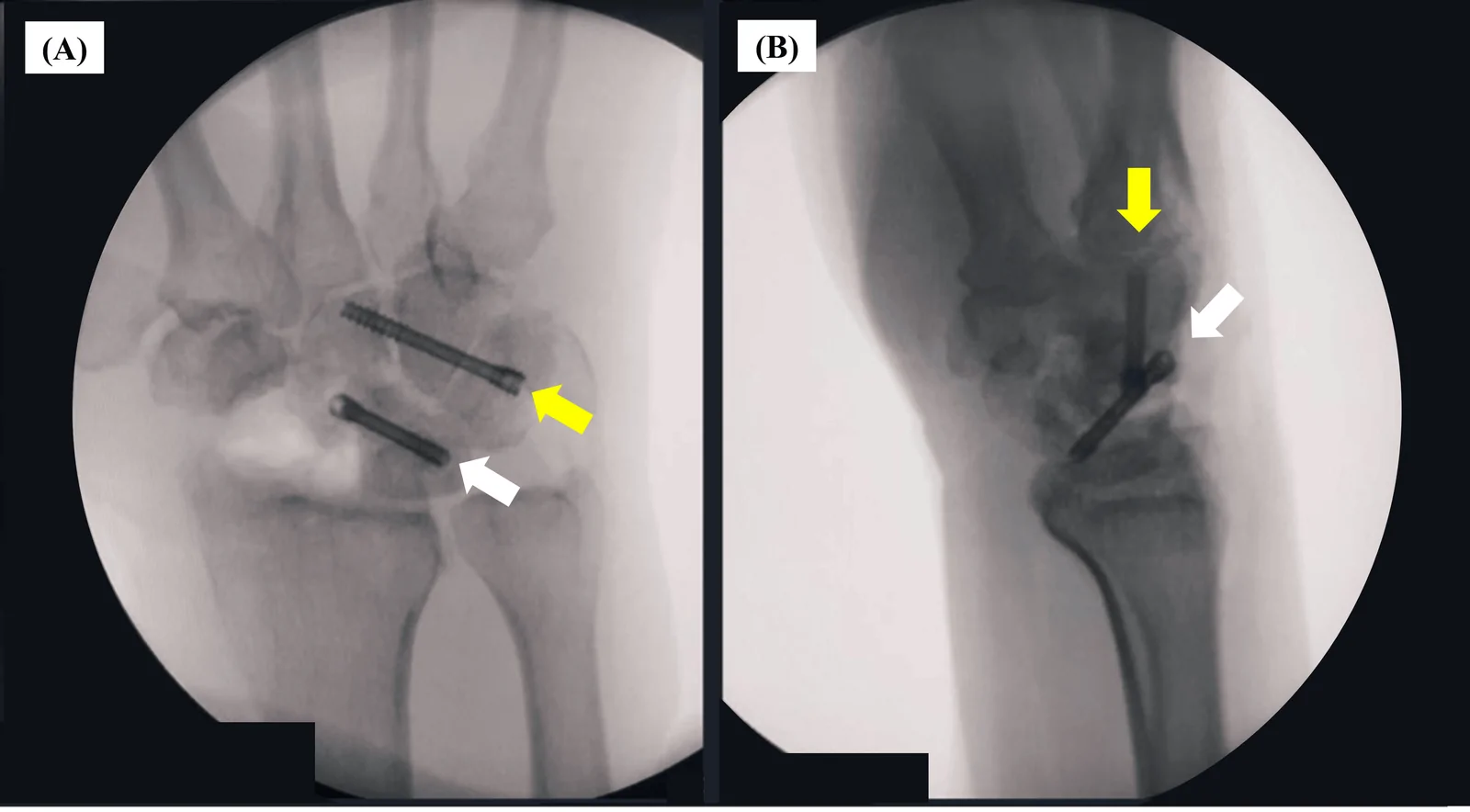
Intraoperative fluoroscopy showing HCS placement in the horizontal configuration group Posteroanterior (A) and lateral (B) fluoroscopic views demonstrate anatomical carpal reduction and stable carpal fixation. The white arrow indicates the HCS placed from the capitate into the lunate. The yellow arrow indicates the transverse screw crossing from the triquetrum through the hamate toward the capitate. HCS: headless compression screw Image Credit: Created by the authors using PowerPoint (Microsoft Corp., Redmond, WA, USA)

Outcome assessment

The primary outcome was time to radiographic union (days), measured from the date of surgery to the date of radiographic union. Radiographic union was defined as trabecular bridging on two standardized wrist radiographs (posteroanterior and lateral views). Secondary endpoints comprised (1) radiographic union rate; (2) preservation of carpal height and radiolunate joint space, assessed using postoperative CHR and JSHR, respectively (Figure 4); and (3) postoperative complications, including hardware failure, infection, neurovascular injury, nonunion, and reoperation. The CHR was calculated as carpal height divided by third metacarpal length, while the JSHR was defined as the radiolunate joint space height divided by carpal height. Both indices were measured on standardized posteroanterior radiographs to evaluate postoperative carpal height maintenance and preservation of the radiolunate joint space.

**Figure 4:**
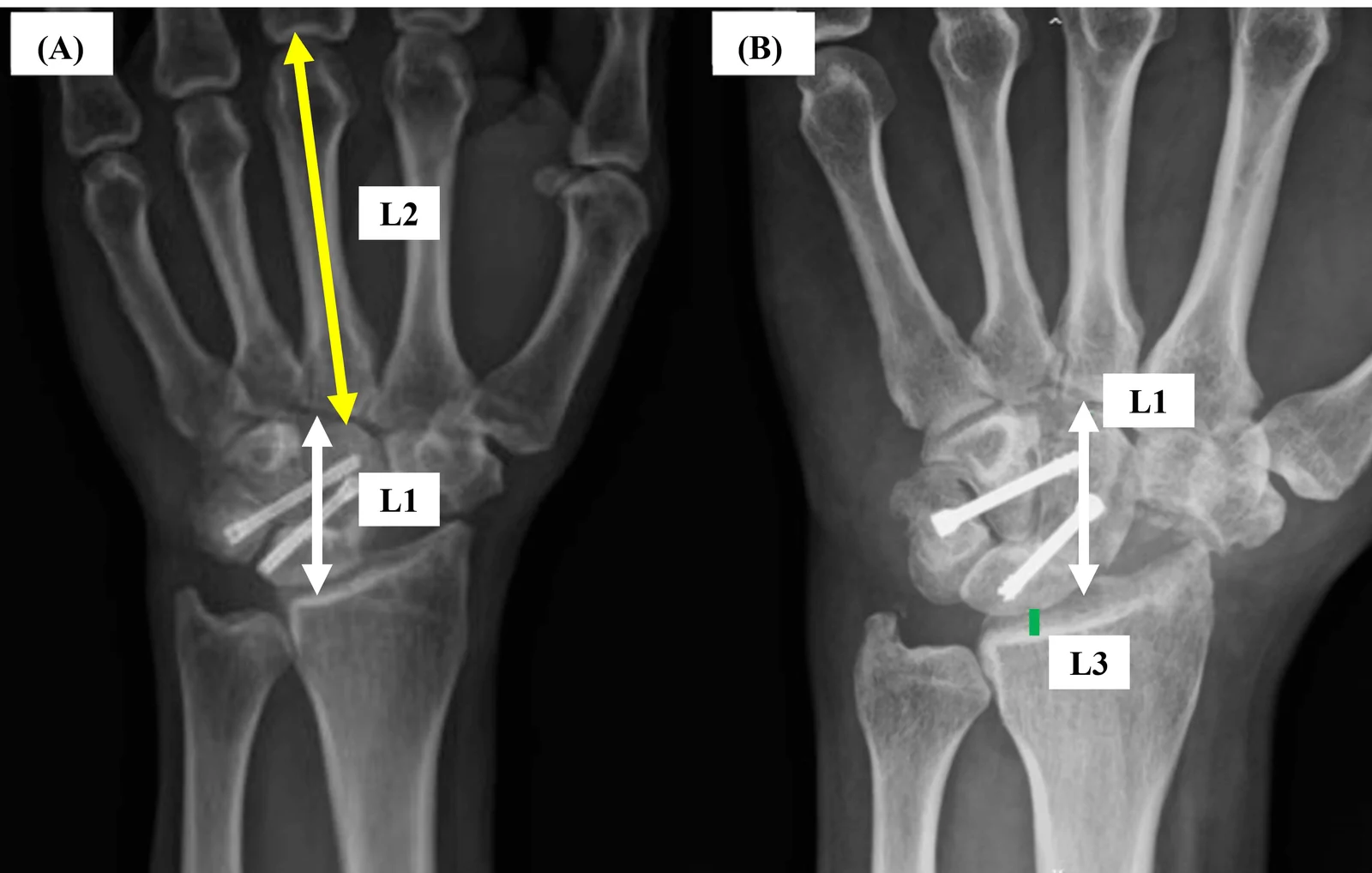
Radiographic assessment of CHR and JSHR (A) CHR measured on a posteroanterior radiograph, calculated as the carpal height (L1, white two-headed arrow; measured from the base of the third metacarpal to the distal radial articular surface) divided by the length of the third metacarpal (L2, yellow two-headed arrow). (B) JSHR measured on a posteroanterior radiograph, calculated as the radiolunate joint space height (L3, green line) divided by the carpal height (L1, white two-headed arrow). CHR: carpal height ratio, JSHR: joint space height ratio Image Credit: Created by the authors using PowerPoint (Microsoft Corp., Redmond, WA, USA)

Statistical analysis

Statistical analyses were performed using Stata version 17 (StataCorp LLC, College Station, TX, USA) [21]. Continuous variables are presented as means ± standard deviations (SD), while categorical variables are presented as frequencies and percentages. Between-group comparisons were performed using the independent-samples t-test for continuous variables and the chi-square test or Fisher's exact test for categorical variables. Within-group changes in CHR and JSHR from preoperative to postoperative assessments were analyzed using the paired t-test or Wilcoxon signed-rank test, depending on the normality of the data distribution as determined by the Shapiro-Wilk test. To support comprehensive data interpretation, relevant numerical test statistics (Student's t and chi-square values) are reported alongside 95% confidence intervals (CIs) and p-values in all comparative analyses. All tests were two-tailed; statistical significance was set at p < 0.05.

## Results

Of the 18 patients initially assessed as meeting the inclusion criteria, two were excluded because of incomplete radiographic records. Sixteen patients were included in the final analysis: eight in the vertical screw configuration group and eight in the horizontal screw configuration group. Baseline demographic and clinical characteristics were comparable between groups (Table 1). The cohort comprised 14 males (87.5%) and two females (12.5%), with a mean age of 49.44 ± 13.01 years. The right upper extremity was involved in 14 patients (87.5%). SNAC wrist was the most common diagnosis (12 patients, 75.0%), and stage III disease was present in 10 patients (62.5%). No statistically significant baseline differences were observed between groups.

**Table 1:** Patient characteristics Data are expressed as mean ± SD for continuous variables and n (%) for categorical variables. Percentages may not total 100% due to rounding. Between-group comparisons were performed using the independent-samples t-test (t) for continuous variables and Fisher's exact test/chi-square test for categorical variables. SD: standard deviation, SNAC: scaphoid nonunion advanced collapse, SLAC: scapholunate advanced collapse, t: Student's t-test statistic, χ²: chi-square test statistic

Characteristics	Total (n = 16)	Horizontal (n = 8)	Vertical (n = 8)	Test statistic	p-value
Sex, n (%)				χ² = 0.00	1.00
Male	14 (87.5)	7 (87.50)	7 (87.50)	-	-
Female	2 (12.5)	1 (12.50)	1 (12.50)	-	-
Age, years	49.44 ± 13.01	51.75 ± 13.40	47.13 ± 12.69	t = 0.71	0.49
Operated hand, n (%)				χ² = 0.00	1.00
Right	14 (87.5)	7 (87.5)	7 (87.5)	-	-
Left	2 (12.5)	1 (12.5)	1 (12.5)	-	-
Type of collapse, n (%)				χ² = 0.00	1.00
SNAC	12 (75.0)	6 (75.0)	6 (75.0)	-	-
SLAC	4 (25.0)	2 (25.0)	2 (25.0)	-	-
Stage of collapse, n (%)				χ² = 0.00	1.00
II	6 (37.5)	3 (37.5)	3 (37.5)	-	-
III	10 (62.5)	5 (62.5)	5 (62.5)	-	-
Follow-up time, days	118.19 ± 61.18	117.88 ± 63.98	118.50 ± 62.72	t = -0.02	0.98

All patients achieved radiographic union, resulting in a union rate of 100.0% in both groups. The mean time to union was 43.48 ± 12.91 days in the vertical screw configuration group and 40.63 ± 11.70 days in the horizontal screw configuration group. The mean difference was 2.85 days (95% CI: -10.42 to 16.12; t = -0.46, p = 0.66), which was not statistically significant (Table 2).

**Table 2:** Comparison of union outcomes between horizontal and vertical screw configurations Data are expressed as mean ± SD for continuous variables and n (%) for categorical variables. Between-group comparisons were performed using the independent-samples t-test for time to union and Fisher’s exact test for union rate. CI: confidence interval, SD: standard deviation, t: Student's t-test statistic, χ²: Chi-square test statistic

Outcome	Horizontal (n = 8)	Vertical (n = 8)	Mean difference (95% CI)	Test statistic	p-value
Time to union, days	40.63 ± 11.70	43.48 ± 12.91	2.85 (-10.42 to 16.12)	t = -0.46	0.66
Union rate, n (%)	8 (100)	8 (100)	0.00	χ² = 0.00	1.00

CHR decreased slightly from preoperative values in both groups but was generally preserved postoperatively. The mean postoperative decrease in CHR was 0.04 ± 0.01 in the horizontal group and 0.03 ± 0.02 in the vertical group. No statistically significant intergroup differences in CHR change magnitude were observed (Table 3). JSHR was also maintained postoperatively in both groups, with no statistically significant difference between groups (Table 4).

**Table 3:** Comparison of CHR between groups Data are expressed as mean ± SD. Between-group comparisons were performed using the independent-samples t-test. CHR: carpal height ratio, CI: confidence interval, SD: standard deviation, t: Student's t-test statistic

CHR	Horizontal (n = 8)	Vertical (n = 8)	Mean difference (95% CI)	Test statistic	p-value
Preoperative CHR	0.49 ± 0.01	0.51 ± 0.07	-0.01 (-0.07 to 0.05)	t = -0.43	0.67
Postoperative CHR	0.46 ± 0.04	0.48 ± 0.07	-0.02 (-0.08 to 0.04)	t = -0.80	0.44
Change in CHR	0.04 ± 0.01	0.03 ± 0.02	0.02 (0.01 to 0.03)	t = 1.04	0.32

**Table 4:** Comparison of JSHR between groups Data are expressed as mean ± SD. Between-group comparisons were performed using the independent-samples t-test. CI: confidence interval, JSHR: joint space height ratio, SD: standard deviation, t: Student's t-test statistic

JSHR	Horizontal (n = 8)	Vertical (n = 8)	Mean difference (95% CI)	Test statistic	p-value
Preoperative JSHR	0.10 ± 0.02	0.10 ± 0.02	0.00 (-0.02 to 0.03)	t = -0.65	0.53
Postoperative JSHR	0.11 ± 0.02	0.10 ± 0.02	0.01 (-0.01 to 0.03)	t = 1.23	0.24
Change in JSHR	-0.01 ± 0.02	0.00 ± 0.02	-0.01 (-0.02 to 0.02)	t = -0.28	0.79

Neither group experienced postoperative complications, including hardware failure, infection, neurovascular injury, nonunion, or reoperation, during the follow-up period. Overall, both vertical and horizontal HCS configurations showed comparable radiographic and union outcomes after FCA in SNAC and SLAC wrists.

## Discussion

This retrospective cohort study compared radiographic outcomes between vertical and horizontal HCS configurations in FCA for stage II-III SNAC and SLAC wrists. Both screw configurations achieved radiographic union in all patients, with no statistically significant differences in time to union, CHR, or JSHR and no reported postoperative complications. These findings support the feasibility and radiographic efficacy of both screw configurations, suggesting that surgeons may select a configuration based on intraoperative anatomy and surgeon preference.

The radiographic union rates in our series mirror those already documented in the literature. In a systematic review of 57 articles, Hayes et al. [18] found an overall reported radiographic union of 97% for all fixation methods, with studies specifically using screw fixation reporting rates approaching 98%. A meta-analysis of eight studies by Andronic et al. [6] evaluating FCA outcomes with long-term follow-up reported an average fusion rate of 91%. Other studies using HCS fixation report union rates between 89% and 100%, again indicating generally positive results [10,12,13,15,19]. All patients in this series achieved radiographic union regardless of screw configuration, consistent with previous reports supporting the effectiveness of HCS fixation in achieving union following FCA.

Given the universally high radiographic union rates reported in the literature following HCS fixation in FCA, union rate alone seems insufficient as a primary outcome measure in comparative studies of screw configuration. We therefore chose time to union as our primary outcome measure, assuming that faster bone bridging would allow earlier rehabilitation and reduce muscle atrophy and soft tissue contracture, thereby improving functional recovery. Previous series have supported this concept. Specifically, Ozyurekoglu and Turker [15], who evaluated the vertical screw configuration, reported a mean time to union of 49 ± 12 days. In contrast, Iordache et al. [19], who used the antegrade horizontal crossed-screw technique, reported a mean time to union of 82 ± 68 days. Differences in time to union across published series support using time to union as a clinically valid, more sensitive primary outcome measure than union rate alone.

In this cohort, the mean difference in time to radiographic union between the horizontal and vertical screw configurations was 2.85 days (95% CI: -10.42 to 16.12; t = -0.46, p = 0.66), with no statistically significant difference observed. Nevertheless, clinicians should interpret this finding cautiously. First, a difference of fewer than three days lies well within the standard four- to six-week follow-up interval commonly employed in hand surgery practice. Second, because no minimal clinically important difference (MCID) has been established for time to union following FCA, it is unclear whether such a modest difference would translate into a noticeable advantage in patient outcomes or function. Quantifying an MCID for time to union would be an ideal goal for future prospective work, because this value guides both clinical significance and study power.

Post hoc power calculations based on the observed mean difference in time to union (2.85 days) and pooled SD (12.3 days) yielded a small effect size (Cohen’s d = 0.23), and a sample size of only eight patients in each group resulted in a power of approximately 6%. At a two-sided alpha level of 0.05, this demonstrates the study's inability to detect small between-group differences in time to union. Thus, the lack of a statistically significant difference suggests preliminary comparability in this small series, not definitive clinical or biomechanical equivalence between the two screw configurations.

Postoperative CHR and JSHR did not differ significantly between the two screw configuration groups. Although CHR decreased modestly in both groups (horizontal: −0.04; vertical: −0.03), this reduction may reflect the expected changes in carpal height following scaphoid excision and midcarpal fusion during FCA rather than fixation failure. These findings are consistent with previously reported changes in carpal height in similar patient populations and may not represent clinically meaningful deterioration [7,10]. The similar postoperative CHR and JSHR findings in both groups suggest that both configurations may provide adequate stability to preserve carpal height and radiolunate joint space.

No patient in either group experienced postoperative complications during follow-up, including hardware failure, implant irritation, infection, neurovascular injury, or reoperation. This finding is particularly notable because previous FCA series have reported nonunion rates of 3-11% and hardware-related irritation requiring removal in 5-15% of patients [7]. Although the absence of these postoperative complications is encouraging, it may be attributable to several factors: (1) meticulous surgical technique; (2) the subchondral placement of HCS, which minimizes dorsal implant prominence; (3) strict adherence to inclusion and exclusion criteria; and (4) the surgical setting of a high-volume tertiary referral center. Previous comparative studies have shown favorable results for HCS fixation. McGinn [14] reported a significantly lower complication rate with HCS fixation than with staple fixation (13% versus 50%). Erne et al. [12] demonstrated greater pain relief, better postoperative Disabilities of the Arm, Shoulder, and Hand (DASH) scores, and shorter operative time with HCS fixation than with K-wire or locking plate fixation. In summary, these results align with the growing evidence supporting HCS fixation as a safe, effective, and technically advantageous alternative for FCA.

This study provides practical guidance for clinical decision-making. To our knowledge, this is the first direct comparison of vertical and horizontal HCS configurations in FCA evaluating time to union as the primary radiographic endpoint. In the absence of direct comparative data, screw orientation has traditionally been guided by surgeon preference, training background, and institutional practices. These findings support an individualized surgical strategy, allowing surgeons to select the screw configuration that best fits the patient's carpal anatomy, bone dimensions, and intraoperative spatial constraints, with no statistically significant differences in the measured radiographic outcomes.

This study has several limitations. The retrospective, nonrandomized design may have introduced selection bias in choosing the screw configuration. The small sample size of 16 patients limits statistical power. The analysis focused only on radiographic outcomes and did not evaluate functional measures such as grip strength, wrist range of motion, pain scores, or validated patient-reported questionnaires, including the DASH questionnaire and the Patient-Rated Wrist Evaluation. The main goal of FCA is to reduce pain and restore function; radiographic comparability alone cannot substantiate therapeutic equivalence [6,10]. Additionally, because the cohort was predominantly male and drawn from a single center with a relatively homogeneous profile, the findings may have limited generalizability. The study did not measure interobserver reliability for radiographic parameters, a potential source of measurement variability. Finally, the mean follow-up duration was approximately 118 days, primarily to document radiographic union and immediate postoperative outcomes. Prospective studies with substantially larger sample sizes and long-term clinical follow-up exceeding two years are needed to evaluate durable functional recovery and progressive degenerative changes.

## Conclusions

No statistically significant differences were detected between vertical and horizontal HCS configurations in FCA regarding time to union, CHR, JSHR, or early postoperative complications in patients with stage II-III SNAC and SLAC wrists. These findings provide preliminary evidence that both configurations may be suitable for different carpal anatomies and surgical preferences. Adequately powered prospective studies with validated functional outcomes and extended follow-up remain necessary.
